# Estimates of eskd risk and timely kidney replacement therapy education

**DOI:** 10.1186/s12882-024-03687-8

**Published:** 2024-09-10

**Authors:** Lauren E. Haggerty, Dena E. Rifkin, Hoang Anh Nguyen, Joseph A. Abdelmalek, Natalie Sweiss, Lindsay M. Miller, O. Alison Potok

**Affiliations:** 1 Division of Nephrology-Hypertension, University of Washington, Seattle, WA USA; 2 Division of Nephrology-Hypertension, University of California, San Diego, CA USA; 3 Veterans Affairs San Diego Healthcare System, San Diego, CA USA; 4 Division of Nephrology-Hypertension, University of California, Irvine, CA USA; 5 Herbert Wertheim School of Public Health and Human Longevity Science, University of California, San Diego, CA USA

**Keywords:** CKD, ESKD risk, Kidney failure, KFRE, Kidney replacement therapy, Education, Dialysis preparation

## Abstract

**Background:**

Kidney replacement therapy (KRT) needs preparation and its timing is difficult to predict. Nephrologists’ predictions of kidney failure risk tend to be more pessimistic than the Kidney Failure Risk Equation (KFRE) predictions. We aimed to explore how physicians’ risk estimate related to referral to KRT education, vs. the objective calculated KFRE.

**Methods:**

Prospective observational study of data collected in chronic kidney disease (CKD) clinics of the Veterans Affairs Medical Center San Diego and the University of California, San Diego. The study included 257 participants who were aged 18 years or older, English speaking, prevalent CKD clinic patients, with estimated glomerular filtration rate (eGFR) < 60 mL/min per 1.73 m^2^ (MDRD equation). The exposure consisted of end stage kidney disease (ESKD) risk predictions. Nephrologists’ kidney failure risk estimations were assessed: “On a scale of 0–100%, without using any estimating equations, give your best estimate of the risk that this patient will need dialysis or a kidney transplant in 2 years.” KFRE was calculated using age, sex, eGFR, serum bicarbonate, albumin, calcium, phosphorus, urine albumin/creatinine ratio. The outcomes were the pattern of referral to KRT education (within 90 days of initial visit) and kidney failure evaluated by chart review. The population was divided into groups either by nephrologists’ predictions or by KFRE. Referral to KRT education was examined by group and sensitivity and specificity were calculated based on whether participants reached kidney failure at 2 years.

**Results:**

A fifth were referred for education by 90 days of enrollment. Low risk patients by both estimates had low referral rates. In those with nephrologists’ predictions ≥ 15% (*n* = 137), sensitivity was 71% and specificity 76%. In those with KFRE ≥ 15% (*n* = 55), sensitivity was 85% and specificity 41%.

**Conclusions:**

Although nephrologists tend to overestimate patients’ kidney failure risk, they do not appear to act on this overestimation, as the rates of KRT education referrals are lower than expected when a nephrologist identifies a patient as high risk.

**Clinical Trial Number:**

Not applicable

**Supplementary Information:**

The online version contains supplementary material available at 10.1186/s12882-024-03687-8.

## Background

Determining when to start preparing chronic kidney disease (CKD) patients for dialysis is one of the most challenging tasks nephrologists have to face. The rate of progression of CKD to kidney failure is non-linear and depends on various factors including the etiology and severity of the kidney disease [1], the presence and degree of proteinuria, the age and comorbidities of the patient, etc. Predicting a patient’s risk of progression to kidney failure is further complicated when considering the risk of patient mortality related to their other comorbidities [2, 3]. If the risk of mortality is higher than that of kidney failure, preparation for kidney replacement therapy (KRT) may be futile.

Overall, the decision to proceed with permanent dialysis access placement should take into account each patient’s comorbidities, risk of complications, and personal preferences and values [4]. The National Kidney Foundation’s Kidney Disease Outcomes Quality Initiative (KDOQI) Guidelines [5] recommend that patients with progressive CKD should be educated on kidney replacement therapy early on, i.e. when estimated glomerular filtration rate (eGFR) reaches 30 mL/min/1.73m^2^ or lower, to allow time for permanent dialysis access placement. Early KRT education is associated with lower hospitalization rates, higher employment rates, and earlier engagement in the transplant process [6].

Despite these recommendations, since the progression of a patient’s disease is difficult to predict, nephrologists and their patients regularly deal with a challenging question: If and when should patients receive dedicated education on kidney replacement modalities? The Kidney Failure Risk Equation (KRFE) [7] is well established as a tool that can reliably predict a patient’s risk of progressing to kidney failure [8–10] in two or five years using either 4 variables demographics (age, sex) and routine laboratory data (eGFR, albuminuria) or else 8 variables (additionally using serum bicarbonate, phosphorus, corrected calcium and albumin). A meta-analysis [11] evaluating cohorts from 30 countries showed that the 2-year KFRE estimation achieved overall excellent discrimination (ability to differentiate those who will reach kidney failure vs. not) with a C-statistic of 0.90 (95% CI [0.89; 0.92]). The KFRE equation includes a calibration factor for non-North American cohorts.

Our previous work [12] revealed that, when comparing the predicted vs. observed risk of kidney failure incidence at 2 years, the 8 variable KFRE outperformed both patients’ and their nephrologists’ estimations of this risk, although all three estimations provided reasonable risk ranking (C-statistics were 0.91, 0.82 and 0.92 for KFRE, patients’ estimates and physicians’ estimates respectively). Patients and physicians overestimated the risk of progression to kidney failure compared to the KFRE, which was both accurate and precise. Here, we aim to examine whether physicians were consistent with their estimations of end stage kidney disease (ESKD) risk in their referral patterns to KRT education, and whether their referrals were appropriate (i.e. referred patients indeed progressed to ESKD).

## Methods

Patients included in this prospective observational study were aged 18 years or older, spoke English, were already established at the clinic (i.e. had been seen in the clinic at least once before), and had an eGFR < 60 mL/min per 1.73 m^2^. Enrollment occurred between July 2015 and June 2016 and participants were followed for 2 years. The KFRE for each patient was calculated using data from the visit when patient was included in the study: age, sex, eGFR (using MDRD equation, in mL/min/1.73 m^2^), serum bicarbonate (mEq/L), albumin (g/dL), calcium (mg/dL), phosphorus (mg/dL), and urine albumin-to-creatinine ratio (mg/g). At the same visit, the nephrologists’ estimations of a patient’s progression to kidney failure were assessed with the following question: “On a scale of 0–100%, without using any estimating equations, please give your best estimate of the risk that this patient will need dialysis or a kidney transplant in 2 years.”

Patients’ timing and rates of referral to KRT classes, as well as incidence of kidney failure and mortality were obtained by chart review. The decision to refer a patient to KRT education was at the discretion of the nephrologist, without access to KFRE scores and separate from physician or patient involvement in the study. Physicians documented referral of patients to kidney replacement therapy classes in the form of ordering the referral in the electronic medical record or explicitly stating referral in their progress notes. The clinic notes were manually reviewed to assess whether patients were referred to classes. Patients were defined as reaching kidney failure if they received a kidney transplant or started dialysis. Death either prior to or after starting KRT was also ascertained based on chart review.

Patients were divided into four groups based on their 2 year KFRE risk as well as their risk of kidney failure as estimated by physicians. The KFRE and physician estimated groups were defined by a risk of developing kidney failure of < 1%; 1-4.9%; 5-14.9%; and ≥ 15%, as per prior work [12]. Since those predictions were made at the initial visit (at time of enrollment), we evaluated patterns of referral to modalities education up to 90 days after the initial visit.

In the high risk groups, we assessed sensitivity as the number of participants who had been referred to KRT education, among those who did reach kidney failure; and specificity as the number of participants who had not been referred among those who did not reach kidney failure.

We performed a sensitivity analysis among those who had not been referred to KRT education prior to enrollment, and who were not lost to follow-up within 2 years.

## Results

**Fig. 1:**
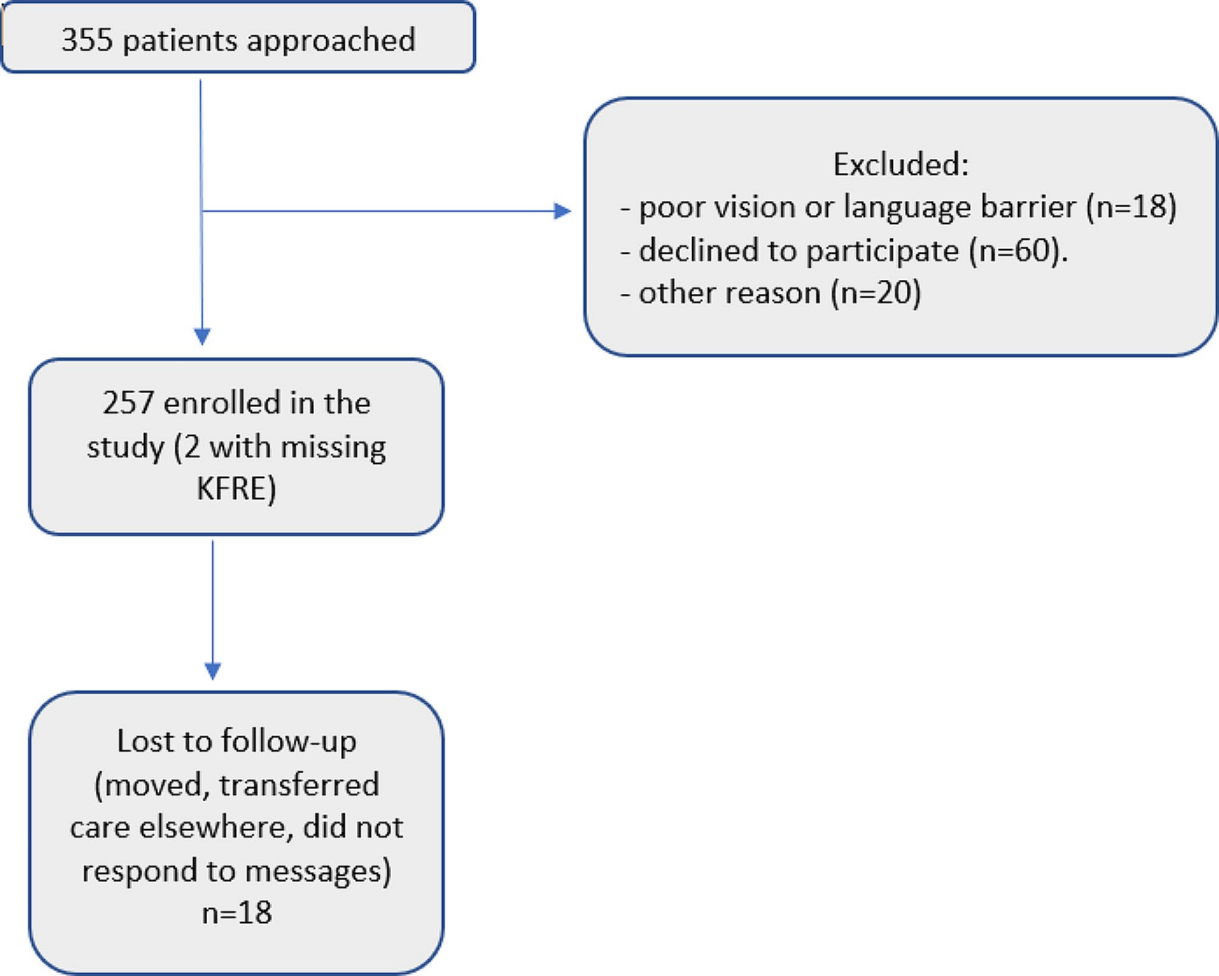
Flow chart diagram

This study included 257 CKD patients (Table 1). The average age was 65 (± 13) years, and eGFR at time of enrollment was 34 (± 13) mL/min/1.73m^2^. About 74% of the patients were men. Thirteen nephrologists participated in this study, seven practiced solely at UCSD, five only at the VA, and one practiced at both sites. They had 14.8 (± 9.4) years of practice on average. The number of participants lost to follow-up was 18 (7%), and this occurred on average 378 days into the study. For the purpose of the analysis, these patients were deemed alive without ESKD. Two participants had missing proteinuria and thus a missing KFRE (Fig. 1).

**Table 1:** Baseline characteristics of participants, divided into groups based on physicians’ ESKD risk estimate

	Physicians’ estimate < 1%	Physicians’ estimate 1-4.9%	Physicians’ estimate 5-14.9%	Physicians’ estimate ≥ 15%
	*n* = 22	*n* = 29	*n* = 69	*n* = 137
Age, years, mean (± SD)	61 (14)	64 (13)	65 (12)	65 (14)
Men, n (%)	12 (55)	23 (79)	50 (72)	105 (77)
Caucasian, n (%)	13 (59)	19 (66)	42 (61)	77 (56)
Black, n (%)	3 (14)	2 (7)	7 (10)	16 (12)
Diabetes, n (%)	2 (9)	13 (45)	30 (43)	77 (56)
Hypertension, n (%)	19 (86)	24 (83)	52 (78)	118 (86)
Baseline eGFR, mL/min/1.73m^2^, mean (± SD)	45 (8)	44 (8)	41 (10)	27 (12)
Physician 2 year estimation of ESKD %(± SD)	0.2% (0.1)	2% (1)	8% (3)	46% (29)
Mean KFRE % (± SD)	1% (1)	1.5% (2)	3% (5)	20% (25)
kidney failure at 2 years, n (%)	0 (0)	0 (0)	0 (0)	34 (25)
Death at 2 years, n (%)	4 (18)	2 (7)	5 (7)	28 (20)
Referred to KRT education, n (%)				
Prior to study enrollment	1 (5)	2 (7)	3 (4)	35 (26)
up to 90 days after study enrollment	1 (5)	2 (7)	3 (4)	49 (36)
Within 2 years	1 (5)	2 (7)	5 (7)	65 (47)

KFRE: Kidney Failure Risk Equation

eGFR: estimated glomerular filtration rate

KRT: kidney replacement therapy

Thirty four of the 257 (13%) participants reached ESKD within 2 years, and 26 (10%) passed away.The median time to ESKD was 257 days (interquartile range, 67–481 days; range, 14–708 days) with a mean (+/- standard deviation) eGFR of 9 (+/-3) mL/min/1.73m^2^. Those who had acute kidney injury requiring dialysis (*n* = 3) during follow-up were not considered to have reached ESKD.

As shown in Table 1, about 21% participants (55/257) were referred for education up to 90 days after enrollment, and 28% (73/257) were referred for KRT education within two years of enrollment. There were higher rates of referral to education prior to or within 90 days after enrollment as estimated kidney failure risk within 2 years increased, examined either by physicians’ estimate or by KFRE.

**Fig. 2:**
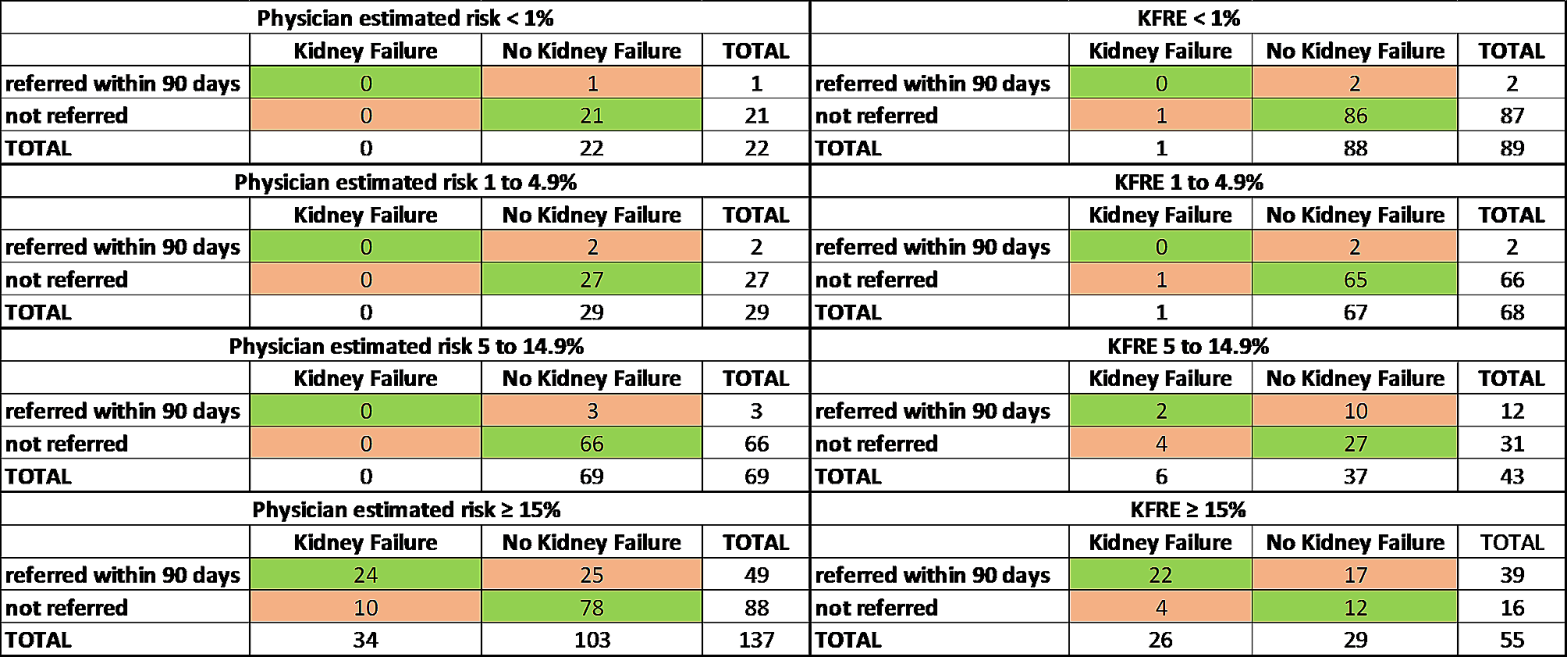
Kidney replacement therapy education referrals by Physicians’ estimates of kidney failure risk and by KFRE at 2 years. KFRE: Kidney Failure Risk Equation. Fisher exact test: *p* < 0.0001 for Physician estimated risk ≥ 15%, *p* = 0.98 for KFRE < 1%, *p* = 0.97 for KFRE 1 to 4.9%, *p* = 0.34 for KFRE 5 to 14.9%, *p* = 0.03 for KFRE ≥ 15%

The rates of referral to KRT education up to 90 days after enrollment were compared to rates of progression to kidney failure at 2 years for low risk patients by both physicians’ estimates and KFRE (Fig. 2). In these groups, rates of referral for education and progression to kidney failure were low. Similar comparisons were described for higher risk groups, where higher rates of referral and kidney failure at 2 years are seen. When nephrologists estimated kidney failure risk as ≥ 15% (*n* = 137), 24 of 34 (71%) of patients who developed kidney failure had been referred within 90 days of enrollment for education, while 78 of 103 (76%) of patients who did not develop kidney failure had not been referred within that time frame (Fisher exact test *p* < 0.0001). Among the 55 participants with a KFRE ≥ 15%, 16 (29%) were not referred to KRT education within 90 days, although 4 of those 16 did reach ESKD (Fisher exact test *p* = 0.03); whereas among those with lower KFRE predictions, the nephrologists referred 16 patients (8%) of whom only 2 progressed to ESKD. In those with KFRE ≥ 15% (*n* = 55), 22 of the 26 (85%) who reached kidney failure had been referred for education (sensitivity). Conversely, only 12 of the 29 (41%) who did not reach kidney failure had not been referred (specificity). (Fig. 2).

When considering the group of patients who progressed to ESKD, all 34 patients had been deemed high risk by their nephrologist (physician’s risk estimation ≥ 15%), 22 of them were referred more than 6 months prior to KRT initiation, while 4 of them were referred less than 6 months prior, and 8 were never referred. Among those who reached ESKD and had a KFRE ≥ 15%, 20 were referred more than 6 months prior to KRT initiation, 2 were referred less than 6 months prior, and 4 were not referred.

Results of the sensitivity analysis are shown in Supplemental Figure S1.

## Discussion

Our study shows that although physicians’ estimations of kidney failure risk tended to be higher than the KFRE, their referral behavior among those they identified as high risk was more judicious than their estimates suggested. In the highest risk group as identified by nephrologists, physician referral to KRT education was somewhat poorly sensitive (71% of those with kidney failure had been referred to KRT education) though more specific (76% of those without kidney failure had not been referred). This finding indicates that while physicians may think a patient is at high risk of progressing to kidney failure, they are not always acting on that estimation by preparing their patient for KRT. Interestingly, although physicians were not provided with the KFRE results during this study, their behavior followed fairly closely on the KFRE model: referral patterns to KRT education for those in the highest risk group as defined by KFRE, were quite sensitive (85%) and poorly specific (41%). Indeed, the KFRE has been shown [12] to be a better calibrated tool for two-year outcomes than physicians’ estimations.

We found that a number of patients with high KFRE or physicians’ estimates were referred to KRT education, although they did not reach kidney failure within a two year period. Patients who attend KRT education have been shown to have better outcomes, higher rates of permanent access, and higher satisfaction with treatment [13–15]. Education programs helping patients understand KRT options are associated with decreased hemodialysis catheter use and increased utilization of home therapy options [16]. Some kidney failure patients report that they would have been more likely to choose an alternate kidney replacement therapy modality if adequately educated in advance [14]. On the other hand, the harms of educating patients who do not ultimately require KRT, include time spent by the patient and the educator and potential undue stress or anxiety for patients. In our previous work [12], we found that many patients stated they were not aware that they had CKD, despite being prevalent patients of the CKD clinic. When referred to KRT education, a non-negligible number of patients are surprised to learn the severity of their disease [17], and the emotion may limit their ability to engage in the training and to recall any information. On the other hand, one could argue that the risks of undergoing KRT education (even if it was not needed) are trivial so that high risk patients should be systematically referred for education. While from the nephrologists’ perspective, a high sensitivity of KRT referral (i.e. high rate of referral among those who reach kidney failure) may be the most important to accomplish, one can imagine that from the patients’ perspective, a high specificity (i.e. high rate of non-referral among those who do not reach kidney failure) is equally important.

This estimation of CKD progression is crucial as the initiation of KRT requires advanced preparation. For those opting for hemodialysis, starting KRT using a catheter has been shown to be associated with poor outcomes such as infection and thrombosis [18]. Ideally, the surgical placement of an arterio-venous fistula (AVF) or graft (AVG) should be done in a timely manner so that there is sufficient time for access to fully mature before initiation of dialysis, and the use of a catheter could be avoided. At the same time, these surgeries are associated with their own risks, including general anesthesia associated risks, bleeding, infection, steal syndrome, and potential failure [19, 20]. This intervention should be avoided if the patient is thought likely not to reach kidney failure, or delayed as much as possible to prevent any unnecessary complications [20].

According to the USRDS database [21], only 18% of patients with kidney failure initiate hemodialysis with a functioning AV fistula or graft. Current barriers to timely permanent access include a lack of formal policies for patient referral, both to a nephrologist and later for surgical access planning including patient education [22–24]. AVF placement is more likely to occur at high volume centers [25] and in centers with formalized pre-dialysis pathways. If nephrologists could more accurately predict a patient’s CKD risk of progression, then perhaps more patients would initiate hemodialysis with permanent access. The use of risk calculators such as the KFRE in the clinical setting may eventually lead to more timely access placement. Here we found that nephrologists are actually starting the process of preparing for KRT in a pattern that aligns more with a KFRE estimation, rather than in a pattern that mirrors their own stated predictions.

The approach taken in this study, through the question “On a scale of 0–100%, without using any estimating equations, give your best estimate of the risk that this patient will need dialysis or a kidney transplant in 2 years,” was meant to reflect ‘real-life’ clinical situations. The estimation of ESKD risk that nephrologists come up with in their mind when seeing a patient is typically not a number, but rather a subjective assessment resulting from information gathered on patient’s history, demographics, physical exam, laboratory and imaging data. Here, we asked physicians to quantify this intuitive risk assessment with a percentage, realizing that the physicians’ answers would be personal and dependent on their own conceptual framework. Each physician’s ESKD risk assessment value was subjective and not standardized. We argue that this subjectivity is an integral part of physicians’ clinical judgment, and while the requested numerical risk estimate may not be directly comparable from one physician to the next, it allows each physician to rank their own patients in terms of who they believe is at higher risk for ESKD.

The strengths of this study include a large sample size with different patient population between two different healthcare systems. To the best of our knowledge, this is the first cohort where physicians’ estimates of risk of kidney failure are directly compared to the KFRE, along with patterns of KRT education referrals, and actual kidney failure outcome at 2 years. Major limitations include the small number of nephrologists surveyed, all working in the same group, making it difficult to generalize the results as other practices may have different referral patterns. The MDRD equation was used to estimate eGFR because it was reported by the lab in both institutions where this study took place. Some participants were referred to KRT education prior to enrollment and we cannot directly assess what the physicians would have predicted as a risk of ESKD at the time of that referral. However, it is reasonable to assume that this risk prediction would likely have been either the same or lower than that at the enrollment visit. Our study did not capture whether the patients actually attended KRT education when referred and within what time frame from the time of referral. No data on estimated death risk [26] was available, although the competing risk of death likely impacted the nephrologists’ estimates of kidney failure risk (if the risk of death prior to reaching kidney failure was felt to be high, a nephrologist may have revised their kidney failure risk estimate downwards).

## Conclusions

As previously established, nephrologists tend to overestimate their patients’ risk of kidney failure. Our new findings indicate that they do not appear to act on this overestimation, as the rates of referral to KRT education are lower than expected when a nephrologist identifies a patient as high risk. Future studies may explore rates of functioning permanent access in relation to risk estimation calculators and patients’ behavior in preparing for dialysis. Our previous work revealed that patients in the present cohort tended to be more optimistic than the KFRE when asked to estimate their risk of progression to kidney failure, yet it is unclear whether patients act on nephrologists’ recommendations by attending KRT education or whether this translates to early access initiation in said patients.

## Electronic supplementary material

Below is the link to the electronic supplementary material.

Supplementary Material 1

## Data Availability

The datasets generated and/or analyzed during the current study are not publicly available but are available from the corresponding author on reasonable request.

## Abbreviations

AVF: Arteriovenous fistula
AVG: Arteriovenous graft
CKD: Chronic kidney disease
eGFR: Estimated glomerular filtration rate
KDOQI: Kidney Disease Outcomes Quality Initiative
KFRE: Kidney failure risk equation
KRT: Kidney replacement therapy
UCSD: University of California San Diego
USRDS: United States Renal Data System
VA: Veterans Affairs
