# Supplementary material for: Estimates of eskd risk and timely kidney replacement therapy education
Source: BMC Nephrol. 2024 Sep 10;25:300. doi: 10.1186/s12882-024-03687-8 (PMC11384691; doi:10.1186/s12882-024-03687-8)
Supplement: Supplementary file 1 — Supplementary Material 1 [file 12882_2024_3687_MOESM1_ESM.pdf]

Supplemental Figure S1. Kidney replacement therapy education referrals by Physicians' estimates of kidney failure risk and by KFRE at 2 years, after excluding those referred prior to enrollment and those lost to follow-up within 2 years (Sensitivity analysis).

Physician estimated risk < 1%				KFRE < 1%			
	Kidney Failure	No Kidney Failure	TOTAL		Kidney Failure	No Kidney Failure	TOTAL
referred within 90 days	0	0	0	referred within 90 days	0	0	0
not referred	0	20	20	not referred	1	78	79
TOTAL	0	20	20	TOTAL	1	78	79
Physician estimated risk 1 to 4.9%				KFRE 1 to 4.9%			
	Kidney Failure	No Kidney Failure	TOTAL		Kidney Failure	No Kidney Failure	TOTAL
referred within 90 days	0	0	0	referred within 90 days	0	0	0
not referred	0	24	24	not referred	1	61	62
TOTAL	0	24	24	TOTAL	1	61	62
Physician estimated risk 5 to 14.9%				KFRE 5 to 14.9%			
	Kidney Failure	No Kidney Failure	TOTAL		Kidney Failure	No Kidney Failure	TOTAL
referred within 90 days	0	0	0	referred within 90 days	1	3	4
not referred	0	58	58	not referred	4	24	28
TOTAL	0	58	58	TOTAL	5	27	32
Physician estimated risk ≥ 15%				KFRE ≥ 15%			
	Kidney Failure	No Kidney Failure	TOTAL		Kidney Failure	No Kidney Failure	TOTAL
referred within 90 days	7	6	13	referred within 90 days	6	3	9
not referred	10	74	84	not referred	4	11	15
TOTAL	17	80	97	TOTAL	10	14	24

KFRE: Kidney Failure Risk Equation

Fisher exact test $p<0.01$ for Physician estimated risk ≥15%, $p=0.41$ for KFRE 5 to 14.9%, $p= 0.06$ for KFRE ≥15%
